# 5-(Phenyl­diazen­yl)tropolone

**DOI:** 10.1107/S1600536812008677

**Published:** 2012-03-03

**Authors:** Tania N. Hill, Moeketsi S. Mangwaela, Gideon Steyl

**Affiliations:** a Department of Chemistry, University of the Free State, PO Box 339, Bloemfontein 9300, South Africa

## Abstract

The title compound [systematic name: (*E*)-2-hy­droxy-5-(phenyl­diazen­yl)cyclo­hepta-2,4,6-trien-1-one], C_13_H_10_N_2_O_2_, is essentially planar with an r.m.s. deviation of 0.036 (2) Å and a dihedral angle of 1.57 (8)° between the phenyl and tropolone rings. In the crystal, mol­ecules are linked by pairs of O—H⋯O hydrogen bonds into inversion dimers. The dimers are further connected by C—H⋯O hydrogen bonds and π–π stacking inter­actions, with centroid–centroid distances of 3.6934 (9) and 3.6282 (9) Å.

## Related literature

For synthetic background, see: Gao & Zheng (2001 ▶). For applications of azo-substituted tropolones, see: Mori *et al.* (2002 ▶). For related systems, see: Shimanouchi & Sasada (1973 ▶); Steyl & Roodt (2006 ▶). For a description of the Cambridge Structural Database, see: Allen (2002 ▶).
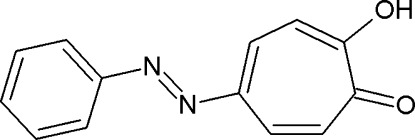


## Experimental

### 

#### Crystal data

C_13_H_10_N_2_O_2_
*M* *_r_* = 226.23
Monoclinic, 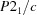

*a* = 6.2838 (2) Å
*b* = 24.8474 (13) Å
*c* = 8.0478 (3) Å
β = 122.255 (2)°
*V* = 1062.64 (8) Å^3^
*Z* = 4
Mo *K*α radiation
μ = 0.10 mm^−1^
*T* = 100 K
0.35 × 0.32 × 0.05 mm

#### Data collection

Bruker X8 APEXII 4K KappaCCD diffractometer
Absorption correction: multi-scan (*SADABS*; Bruker, 2004 ▶) *T*_min_ = 0.967, *T*_max_ = 0.995
10621 measured reflections
2669 independent reflections
1925 reflections with *I* > 2σ(*I*)
*R*_int_ = 0.050

#### Refinement

*R*[*F* ^2^ > 2σ(*F* ^2^)] = 0.048
*wR*(*F* ^2^) = 0.128
*S* = 1.05
2669 reflections
155 parameters
H-atom parameters constrained
Δρ_max_ = 0.38 e Å^−3^
Δρ_min_ = −0.24 e Å^−3^

### 

Data collection: *APEX2* (Bruker, 2005 ▶); cell refinement: *SAINT-Plus* (Bruker, 2004 ▶); data reduction: *SAINT-Plus*; program(s) used to solve structure: *SHELXS97* (Sheldrick, 2008 ▶); program(s) used to refine structure: *SHELXL97* (Sheldrick, 2008 ▶); molecular graphics: *DIAMOND* (Brandenburg & Putz, 2005 ▶); software used to prepare material for publication: *WinGX* (Farrugia, 1999 ▶).

## Supplementary Material

Crystal structure: contains datablock(s) global, I. DOI: 10.1107/S1600536812008677/is5073sup1.cif

Structure factors: contains datablock(s) I. DOI: 10.1107/S1600536812008677/is5073Isup2.hkl

Supplementary material file. DOI: 10.1107/S1600536812008677/is5073Isup3.cml

Additional supplementary materials:  crystallographic information; 3D view; checkCIF report

## Figures and Tables

**Table 1:** Hydrogen-bond geometry (Å, °)

*D*—H⋯*A*	*D*—H	H⋯*A*	*D*⋯*A*	*D*—H⋯*A*
O1—H1⋯O2	0.84	2.08	2.5754 (15)	117
C5—H5⋯O1^i^	0.95	2.40	3.1866 (19)	140
O1—H1⋯O2^ii^	0.84	1.96	2.6686 (15)	141

Symmetry codes: (i) 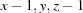; (ii) 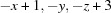.

**Fig. 1.**
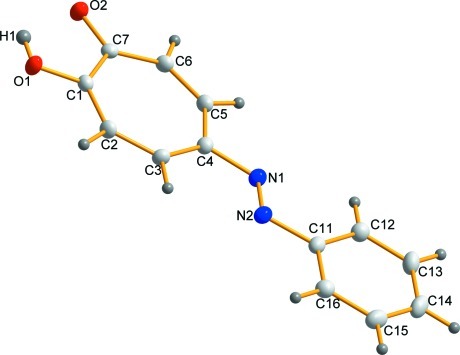
Molecular structure of the title compound (50% probability displacement ellipsoids).

**Fig. 2.**
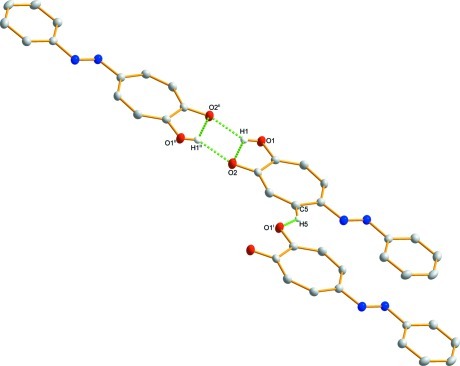
Intermolecular hydrogen bond interactions (dashed bonds) of the title compound. Symmetry codes: (i) x - 1, y, z - 1 (ii) 1 - x, -y, 3 - z. Non-relevant hydrogen atoms have been omitted for clarity.

**Fig. 3.**
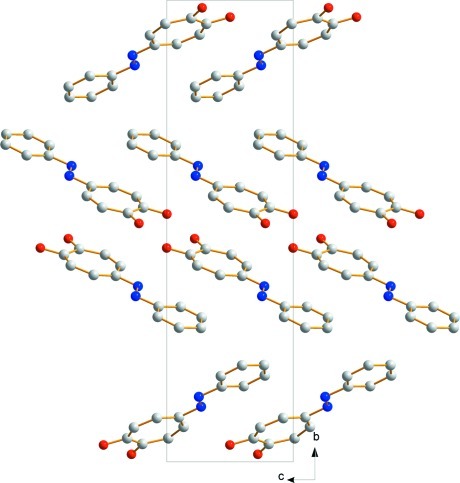
A packing diagram of the title compound, illustrating the parallel sheet configuration as viewed along the a axis.

**Fig. 4.**
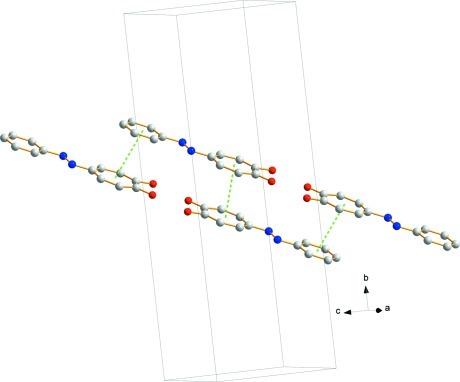
Partially filled unit cell of the title compound, illustrating π–π stacking interactions. Hydrogen atoms have been omitted for clarity.

## supplementary crystallographic information

### Comment

Due to the special nature of the tropolone ring it demonstrates similar aromaticity as the benzene ring, undergoing electrophilic substitution reactions with electrophilic reagents. As part of our study on the functionlization of the tropolone moeity we report the structure of the title compound, (I) (Fig. 1), with the aim of contributing to a deeper understanding of troponoids and its functionlization. The original tropolone crystal structure was done by Shimanouchi & Sasada (1973). A search of the Cambridge structural database (CSD) (Allen, 2002) yielded thirteen troponoid compounds with a mono-substituted 5-position, of these only seven were with the tropolone backbone, none of which had an azo linking group.

In I the dihedral angle between the least-squares planes A (O1/O2/C1–C7/N1) and B (N2/C11–C16) was found to be 1.41 (6)°, resulting in an almost planar molecule with an *r.m.s.* deviation of 0.036 (2) Å. The largest variance from the molecular plane was found to be the O1 atom with a value of 0.058 (1) Å. The well known O—H···H interactions found for tropolone are present and lead to the formation of centrosymmetric dimers (Fig. 2). These interactions along with the last interaction found in Fig. 2, that of tropolone (C5) with an adjacent tropolone (O2) results in the formation of a planar sheet packing configuration (Fig 3). π–π Interactions were observed between the phenyl ring and the tropolone ring with a distance of 3.6934 (9) Å and two tropolone rings with a distance of 3.6282 (9) Å (Fig. 4).

### Experimental

Sodium nitrite (1.4 mmol) dissolved in water (1 ml) was added dropwise to a solution containing aniline (1.6 mmol), hydrochloric acid (2 ml, conc) and water (7 ml). Upon cooling the resultant mixture to *ca.* 4 °C it was slowly added to a solution of sodium hydroxide (1.8 mmol), tropolone (1.6 mmol) in water (4 ml) keeping the temperature < 5 °C. The resulting solution was stirred for 30 minutes, filtered and air-dried. Crystals suitable for X-ray diffraction were obtained by recrystalization with CHCl_3_.

### Refinement

All H atoms were placed in geometrically idealized positions and constrained to ride on their parent atoms.

### Crystal data

**Table:**

C_13_H_10_N_2_O_2_	*F*(000) = 472
*M**_r_* = 226.23	*D*_x_ = 1.414 Mg m^−^^3^
Monoclinic, *P*2_1_/*c*	Mo *K*α radiation, λ = 0.71073 Å
Hall symbol: -P 2ybc	Cell parameters from 1666 reflections
*a* = 6.2838 (2) Å	θ = 3.3–24.9°
*b* = 24.8474 (13) Å	µ = 0.10 mm^−^^1^
*c* = 8.0478 (3) Å	*T* = 100 K
β = 122.255 (2)°	Plate, red
*V* = 1062.64 (8) Å^3^	0.35 × 0.32 × 0.05 mm
*Z* = 4	

### Data collection

**Table:**

Bruker X8 APEXII 4K KappaCCD diffractometer	2669 independent reflections
Radiation source: sealed tube	1925 reflections with *I* > 2σ(*I*)
Graphite monochromator	*R*_int_ = 0.050
Detector resolution: 512 pixels mm^-1^	θ_max_ = 28.4°, θ_min_ = 3.1°
φ and ω scans	*h* = −8→8
Absorption correction: multi-scan (*SADABS*; Bruker, 2004)	*k* = −31→32
*T*_min_ = 0.967, *T*_max_ = 0.995	*l* = −10→10
10621 measured reflections	

### Refinement

**Table:**

Refinement on *F*^2^	0 restraints
Least-squares matrix: full	H-atom parameters constrained
*R*[*F*^2^ > 2σ(*F*^2^)] = 0.048	*w* = 1/[σ^2^(*F*_o_^2^) + (0.0566*P*)^2^ + 0.2818*P*] where *P* = (*F*_o_^2^ + 2*F*_c_^2^)/3
*wR*(*F*^2^) = 0.128	(Δ/σ)_max_ < 0.001
*S* = 1.05	Δρ_max_ = 0.38 e Å^−^^3^
2669 reflections	Δρ_min_ = −0.24 e Å^−^^3^
155 parameters	

### Special details

**Table:**

Geometry. All e.s.d.'s (except the e.s.d. in the dihedral angle between two l.s. planes) are estimated using the full covariance matrix. The cell e.s.d.'s are taken into account individually in the estimation of e.s.d.'s in distances, angles and torsion angles; correlations between e.s.d.'s in cell parameters are only used when they are defined by crystal symmetry. An approximate (isotropic) treatment of cell e.s.d.'s is used for estimating e.s.d.'s involving l.s. planes.

### Fractional atomic coordinates and isotropic or equivalent isotropic displacement parameters (Å^2^)

**Table:**

	*x*	*y*	*z*	*U*_iso_*/*U*_eq_	
C1	0.6923 (3)	0.05118 (6)	1.3134 (2)	0.0163 (3)	
C2	0.8463 (3)	0.07450 (7)	1.2597 (2)	0.0179 (3)	
H2	1.0185	0.077	1.3622	0.022*	
C3	0.7891 (3)	0.09498 (7)	1.0787 (2)	0.0175 (3)	
H3	0.9269	0.1095	1.0758	0.021*	
C4	0.5591 (3)	0.09684 (7)	0.9038 (2)	0.0172 (3)	
C5	0.3285 (3)	0.07598 (7)	0.8670 (2)	0.0177 (3)	
H5	0.1911	0.08	0.7352	0.021*	
C6	0.2696 (3)	0.05111 (7)	0.9882 (2)	0.0181 (4)	
H6	0.099	0.04	0.9262	0.022*	
C7	0.4217 (3)	0.03894 (6)	1.1931 (2)	0.0163 (3)	
C11	0.6741 (3)	0.16352 (6)	0.5654 (2)	0.0178 (3)	
C12	0.4382 (3)	0.16419 (7)	0.3910 (2)	0.0209 (4)	
H12	0.2969	0.1489	0.3868	0.025*	
C13	0.4112 (3)	0.18728 (7)	0.2241 (2)	0.0247 (4)	
H13	0.251	0.1878	0.105	0.03*	
C14	0.6179 (3)	0.20974 (7)	0.2304 (2)	0.0258 (4)	
H14	0.5989	0.2255	0.1158	0.031*	
C15	0.8514 (3)	0.20909 (7)	0.4036 (3)	0.0252 (4)	
H15	0.9922	0.2246	0.4076	0.03*	
C16	0.8811 (3)	0.18585 (7)	0.5718 (2)	0.0213 (4)	
H16	1.0418	0.1852	0.6904	0.026*	
N1	0.5284 (2)	0.11965 (6)	0.72852 (19)	0.0194 (3)	
N2	0.7201 (3)	0.14104 (6)	0.74631 (18)	0.0196 (3)	
O1	0.8030 (2)	0.03666 (5)	1.50085 (15)	0.0204 (3)	
H1	0.6956	0.0231	1.5202	0.031*	
O2	0.3290 (2)	0.01652 (5)	1.27961 (15)	0.0219 (3)	

### Atomic displacement parameters (Å^2^)

**Table:**

	*U*^11^	*U*^22^	*U*^33^	*U*^12^	*U*^13^	*U*^23^
C1	0.0157 (8)	0.0170 (8)	0.0138 (7)	0.0017 (6)	0.0064 (6)	−0.0002 (6)
C2	0.0134 (8)	0.0216 (9)	0.0144 (7)	−0.0009 (6)	0.0046 (6)	−0.0004 (6)
C3	0.0162 (8)	0.0195 (9)	0.0185 (8)	−0.0022 (6)	0.0103 (7)	−0.0004 (6)
C4	0.0185 (8)	0.0174 (9)	0.0151 (7)	−0.0002 (6)	0.0087 (6)	−0.0003 (6)
C5	0.0154 (8)	0.0204 (9)	0.0142 (7)	0.0007 (6)	0.0058 (6)	−0.0006 (6)
C6	0.0139 (7)	0.0218 (9)	0.0162 (7)	−0.0007 (6)	0.0065 (6)	−0.0013 (6)
C7	0.0160 (8)	0.0170 (9)	0.0176 (7)	0.0008 (6)	0.0101 (6)	−0.0010 (6)
C11	0.0223 (8)	0.0149 (8)	0.0186 (8)	0.0008 (6)	0.0125 (7)	0.0004 (6)
C12	0.0217 (8)	0.0198 (9)	0.0214 (8)	−0.0015 (7)	0.0115 (7)	0.0003 (6)
C13	0.0296 (9)	0.0216 (10)	0.0192 (8)	0.0011 (7)	0.0105 (7)	0.0037 (7)
C14	0.0387 (10)	0.0183 (9)	0.0244 (9)	−0.0006 (8)	0.0195 (8)	0.0037 (7)
C15	0.0315 (10)	0.0197 (9)	0.0324 (9)	−0.0029 (7)	0.0225 (8)	0.0001 (7)
C16	0.0223 (8)	0.0209 (9)	0.0218 (8)	−0.0001 (7)	0.0125 (7)	−0.0009 (7)
N1	0.0182 (7)	0.0218 (8)	0.0182 (7)	−0.0015 (6)	0.0097 (6)	0.0009 (5)
N2	0.0204 (7)	0.0213 (8)	0.0188 (7)	−0.0005 (6)	0.0116 (6)	0.0004 (6)
O1	0.0152 (6)	0.0285 (7)	0.0151 (5)	−0.0033 (5)	0.0065 (5)	0.0033 (5)
O2	0.0162 (6)	0.0306 (7)	0.0185 (6)	−0.0019 (5)	0.0089 (5)	0.0036 (5)

### Geometric parameters (Å, º)

**Table:**

C1—O1	1.3301 (18)	C11—C16	1.389 (2)
C1—C2	1.380 (2)	C11—C12	1.395 (2)
C1—C7	1.471 (2)	C11—N2	1.4378 (19)
C2—C3	1.397 (2)	C12—C13	1.385 (2)
C2—H2	0.95	C12—H12	0.95
C3—C4	1.379 (2)	C13—C14	1.389 (2)
C3—H3	0.95	C13—H13	0.95
C4—C5	1.414 (2)	C14—C15	1.383 (2)
C4—N1	1.4342 (19)	C14—H14	0.95
C5—C6	1.362 (2)	C15—C16	1.390 (2)
C5—H5	0.95	C15—H15	0.95
C6—C7	1.429 (2)	C16—H16	0.95
C6—H6	0.95	N1—N2	1.2530 (19)
C7—O2	1.2521 (18)	O1—H1	0.84
			
O1—C1—C2	116.06 (13)	C16—C11—C12	120.25 (14)
O1—C1—C7	114.31 (13)	C16—C11—N2	116.06 (14)
C2—C1—C7	129.62 (14)	C12—C11—N2	123.69 (14)
C1—C2—C3	130.28 (14)	C13—C12—C11	119.66 (15)
C1—C2—H2	114.9	C13—C12—H12	120.2
C3—C2—H2	114.9	C11—C12—H12	120.2
C4—C3—C2	128.53 (15)	C12—C13—C14	120.16 (16)
C4—C3—H3	115.7	C12—C13—H13	119.9
C2—C3—H3	115.7	C14—C13—H13	119.9
C3—C4—C5	126.89 (14)	C15—C14—C13	120.03 (15)
C3—C4—N1	122.36 (14)	C15—C14—H14	120
C5—C4—N1	110.72 (13)	C13—C14—H14	120
C6—C5—C4	131.02 (15)	C14—C15—C16	120.34 (16)
C6—C5—H5	114.5	C14—C15—H15	119.8
C4—C5—H5	114.5	C16—C15—H15	119.8
C5—C6—C7	130.70 (15)	C11—C16—C15	119.55 (15)
C5—C6—H6	114.7	C11—C16—H16	120.2
C7—C6—H6	114.7	C15—C16—H16	120.2
O2—C7—C6	120.84 (14)	N2—N1—C4	116.00 (13)
O2—C7—C1	116.31 (14)	N1—N2—C11	112.83 (13)
C6—C7—C1	122.85 (14)	C1—O1—H1	109.5
			
O1—C1—C2—C3	−178.41 (16)	C16—C11—C12—C13	0.0 (2)
C7—C1—C2—C3	2.7 (3)	N2—C11—C12—C13	179.29 (15)
C1—C2—C3—C4	−0.4 (3)	C11—C12—C13—C14	−0.1 (3)
C2—C3—C4—C5	−2.9 (3)	C12—C13—C14—C15	0.0 (3)
C2—C3—C4—N1	179.51 (16)	C13—C14—C15—C16	0.3 (3)
C3—C4—C5—C6	2.2 (3)	C12—C11—C16—C15	0.3 (2)
N1—C4—C5—C6	−179.90 (17)	N2—C11—C16—C15	−179.08 (15)
C4—C5—C6—C7	1.3 (3)	C14—C15—C16—C11	−0.4 (3)
C5—C6—C7—O2	178.42 (17)	C3—C4—N1—N2	−4.4 (2)
C5—C6—C7—C1	−2.1 (3)	C5—C4—N1—N2	177.61 (14)
O1—C1—C7—O2	−0.2 (2)	C4—N1—N2—C11	−179.34 (13)
C2—C1—C7—O2	178.73 (16)	C16—C11—N2—N1	−177.26 (14)
O1—C1—C7—C6	−179.64 (14)	C12—C11—N2—N1	3.4 (2)
C2—C1—C7—C6	−0.7 (3)		

### Hydrogen-bond geometry (Å, º)

**Table:**

*D*—H···*A*	*D*—H	H···*A*	*D*···*A*	*D*—H···*A*
O1—H1···O2	0.84	2.08	2.5754 (15)	117
C5—H5···O1^i^	0.95	2.40	3.1866 (19)	140
O1—H1···O2^ii^	0.84	1.96	2.6686 (15)	141

Symmetry codes: (i) *x*−1, *y*, *z*−1; (ii) −*x*+1, −*y*, −*z*+3.
